# Antinociceptive and Anti-Inflammatory Effects of *Nypa fruticans* Wurmb by Suppressing TRPV1 in the Sciatic Neuropathies

**DOI:** 10.3390/nu12010135

**Published:** 2020-01-03

**Authors:** Mi-sun Kang, Kyung-Yae Hyun

**Affiliations:** 1 Department of Rehabilitation medicine, Pusan National University Yangsan Hospital, Yangsan 50612, Korea; misunpnuyh@gmail.com; 2 Department of Clinical Laboratory Science, Dong-Eui University, Busan 47340, Korea

**Keywords:** *Nypa fruticans* Warmb (NF), antinociceptive, anti-inflammatory, sciatic neuropathies

## Abstract

Neuropathic pain is generally characterized by sensory abnormalities such as sensory disorders, hyperalgesia, and allodynia. Recent studies have reported that TRPV1 activation is essential for establishing of inflammation in the neuropathy pain models, showing that the expression of this receptor is increased, and contributing to enhanced thermal sensitivity. *Nypa fruticans* Wurmb (NF), which was used as a folk remedy, is a plant that is gaining attention due to its various effects. In this study, we investigated the antinociceptive and anti-inflammatory effects of NFE (*Nypa fruticans* Wurmb extracts) by controlling the neurological function of TRPV1. In sciatic crush injury rat models, a significant level of antinociceptive effect was observed in the thermal hyperalgesia test in which NF extracts (NFE 500 mg/kg) were orally administered, daily. Protein quantification of the sciatic nerve and the of the L4–L6 spinal cord showed a decrease of the TRPV1 expression, the inflammatory expression factor, COX2, and proinflammatory factors in the NFE treated groups. Our results indicate that NFE affects antinociceptive and anti-inflammatory by controlling TRPV1 in sciatic neuropathic pain models.

## 1. Introduction

Neuropathic pain is generally characterized by sensory abnormalities such as sensory disorders, hyperalgesia, and allodynia. Additionally, inflammation of the central and peripheral nervous system helps to cause and sustain chronic neuropathic pain [1]. Issues related to crushing injuries include increased endoneurial edema, free oxygen radicals, inflammatory reactions, de- and remyelination, axonolysis, regeneration, and degeneration [2,3,4]. Inflammatory mediators such as prostaglandins, histamines, cytokines, chemokines, and growth factors are involved in nerve regeneration and degeneration that is associated with neuropathic pain [5,6,7]. Recent studies have described that TRPV1 activation is essential for establishing inflammation and neuropathy pain models, showing that the expression of this receptor is increased, which contributes to enhanced thermal sensitivity [8,9]. Activation of TRPV1 leads to the release of peptide neurotransmitters such as CGRP and SP of neurons involved in pain transmission and neurogenic inflammatory responses [10,11,12]. Although TRPV1 activation does not completely clarify how inflammatory responses are regulated, TRPV1 activation induces neurogenic inflammation. TRPV1 can modulate the primary and stimulus-induced release of pro-/anti-inflammatory cytokines and alleviate neurological diseases associated with inflammation [13]. Therefore, recent studies have been conducted regarding how to treat neuropathic pain by inhibiting targets such as TNF-α, TRPV1, and COX2 [13]. 

However, there is no definitive treatment for peripheral nerve damage. Many drugs have been used for treating experimentally-induced peripheral nerve injuries, including nonsteroidal anti-inflammatory agents, steroids, nerve growth factors, erythropoietin, thyroid hormone, growth hormone, adrenocorticotropic hormone, and insulin-like peptides [3,14]. Considering that the use of analgesic and anti-inflammatory drugs exerts a wide range of side effects [15], there is currently a keen interest in developing new therapeutic agents from natural products [16]. *Nypa fruticans* Wurmb (NF) is a mangrove plant that grows only on mudflats and salt marshes in Malaysia, Indonesia, Papua New Guinea, the Philippines, and Myanmar. The average length of the leaves is 9–10 m [17]. It is known to be rich in polyphenols, flavonoids, vitamin E, and calcium [17]. Previous studies have shown that polyphenols and flavonoids are abundant, with chlorogenic and protocatechuic acid, as well as kaempferol, being prevalent. These are known to have good antioxidative, anti-inflammatory, and cholesterol-suppressing effects [18]. Folk remedies have used the roots, leaves, and stems of NF for conditions like asthma, tuberculosis, sore throats, liver disease, and as an analgesic [19,20]. 

Previous studies have reported the anti-nociception, antioxidant, and anti-inflammatory effects of NF. However, there have been no *Nypa fruticans* Wurmb extract (NFE) related studies regarding the control of TRPV1 in relation to neuropathic pain. Therefore, this study was carried out with the hypothesis that NFE has an anti-nociceptive and anti-inflammatory effect by controlling the neurological function of TRPV1.

## 2. Materials and Methods

### 2.1. Experimental Animal

Male Sprague–Dawley rats (Hyochang science, Daegu, Korea) were used at 4 weeks of age (body weight 100–120 g). During the experiment, water and food (solid feed) were supplied unlimitedly. The temperature of the feed room was maintained at 20 ± 2 °C and the humidity 55 ± 5%. Experiments were carried out after one week of incubation period adaptation period. 

### 2.2. Preparation of the Extract

NF flower stalk used in this experiment were collected in Myanmar, peeled locally, dried, and then purchased commercially available products imported and distributed by Todifarm Korea (Hanam City, Korea). The dried NF was ground using a pulverizer, after which NF powder 100 g extracted with 1 L of 80% EtOH at room temperature for 4 h. Following this, the filter cake was used to remove the powder, the filtered liquid concentrated with an evaporator and dried. The extract was stored at −20 °C in sterile universal bottles.

### 2.3. Sciatic Nerve Crush Injury Model

All experimental procedures were performed after approval by the Animal Experiment Ethic Committee of Dong-Eui University (R2018-002) in accordance with the guidelines of the International Association for the Study of Pain (IASP). The rat was treated with 300 mg/kg of Tribromoethanol (Avertin) intraperitoneally before surgical treatment. The rat underwent general anesthesia with a 2 cm incision of the skin on its right posterior femur, minimizing the damage to the surrounding tissues. Hemostatic forceps were used to crush the exposed sciatic nerve for 30 s according to the modified procedure described by Kalender, A [21]. The wound was then sutured, and the skin was sterilized to prevent infection. The wound was stabilized by putting the rat in a cage for experimental animals [21]. 

### 2.4. Experimental Group and Medication

The Sham group was defined as the group that did not undergo any treatment. Male Sprague–Dawley rats were administered NFE or vehicle or aspirin once daily. Oral gavage using a 20 gauge curved dosing needles are used. The experimental group was treated with NFE 500 mg/kg orally, once daily following the sciatic nerve crush injury (CNI). The aspirin (200 mg/kg) treated group, a positive control, was orally administered aspirin once daily following the CNI. The SB-366791 (Enzo Life Sciences, Farmingdale, NY, USA) as the TRPV1 antagonist was intraperitoneally injected once daily with 0.3 mg/kg. SB366791 was purchased from Enzo (Farmingdale, NY, USA) and was dissolved in 100% ethanol (10 mg/mL with warming) as well as diluted in physiological saline. Aspirin was dissolved in physiological saline. SB366791 [*N*-(3-methoxyphenyl)-4-chlorocinnamide] is a more selective and in vivo is also a more potent TRPV1 antagonist than the more commonly used TRPV1 antagonist capsazepine [22]. It has been widely used as a selective TRPV1 antagonist in pain research [23,24,25].

Sham: Intact rats + saline (10 mL/kg)

Vehicle: Sciatic crush nerve injury (CNI) + saline (10 mL/kg)

Aspirin: Sciatic crush nerve injury (CNI) + aspirin (200 mg/kg)

NFE: Sciatic crush nerve injury (CNI) + NFE (500 mg/kg)

SB: Sciatic crush nerve injury (CNI) + SB366791 (0.3 mg/kg)

NF + SB: Sciatic crush nerve injury (CNI) + NFE (500 mg/kg) + SB366791 (0.3 mg/kg)

### 2.5. Heat Hyperalgegia Test

An evaluation method to measure the pain threshold of the heat was used. The paw withdrawal latency was measured at the 0th, 3rd, 7th, 10th, and 14th day after the sciatic nerve injury by immersing the right hind limb of the sciatic nerve-injured rat in hot water (46 °C). To prevent tissue damage, measurements were taken within 20 s and repeated 3 times at 5 min intervals.

### 2.6. Protein Quantification (Western Blotting)

Sciatic nerve and L4-L6 spinal cord were separated and homogenized using a homogenizer at 4 °C with lysis buffer (PRO-Prep™). Homogenized tissue centrifuged at 13,000 rpm for 10 min. The protein concentration was measured at 595 nm by adding Bio-Rad Protein Assay Kit (Bio-Rad Hercules, CA, USA). The prepared sample was subjected to electrophoresis and nitrocellulose membrane protein transfer and COX2 (1:1000, Cell Signaling, Danvers, MA, USA) and TRPV1 (1:1000, Neuromics, Edina, MN, USA) primary antibody were reacted at 4 °C for 24 h, reacted with secondary antibody for 2 h, and then ECL prime (Amersham Pharmacia Biotech, Buckinghamshire, UK). The degree of expression was measured using Image J software.

### 2.7. Analysis of Inflammatory Markers (Cytokine Analysis)

The blood collected from the abdominal vein was stored in the SST tube. SST tube blood specimens were centrifuged at 3000 rpm for 15 min, and the serum was transferred to an E-tube and stored at −70 °C for analysis. IL-6, TNF-α and IL-1β were measured using an ELISA kit (R & D systems, Minneapolis, MN, USA). After washing with 200 μL of washing solution in a 96-well flat-bottom ELISA plate, 300 uL washing solution is dispensed into each well, washed three times, and then the paper towel is wiped off. Serum was added to each well in an amount of 100 μL, and the plate was covered with a plate sealer and reacted at room temperature for 2 h. After washing 4 times with washing solution, 100 μL of detection antibody (0.25 μg/mL) was added to each well, followed by reaction for 30 min, followed by washing with washing solution 4 times. 100 μL color development solution was added to each well and reacted at room temperature for 5 min. The stop reaction was stopped by stopping the enzyme reaction at 100 μL, and the enzyme reaction was terminated. Using a Spectrophotometer (Bio-Tek EL 808, Bio-Tek Instruments Inc., Colmar, France) and the absorbance at 450 nm was measured. Serum concentrations of IL-6, TNF-α, and IL-1β were calculated based on the standard curve. The cytokine levels were expressed in pg/mL in the serum.

### 2.8. Statistical Analysis

All data are presented as the means ± SEM. Statistical procedures were performed using SPSS version 18. For parametric parameters, possible differences were assessed with ANOVA followed by Tukey test for the post-hoc analysis. For non-parametric data, Kruskal–Wallis test with Dunn’s multiple comparison test were used for significant differences. Statistical significance is assumed for *p* < 0.05. 

## 3. Results

### 3.1. Heat Hyperalgegia Test

**Figure 1:**
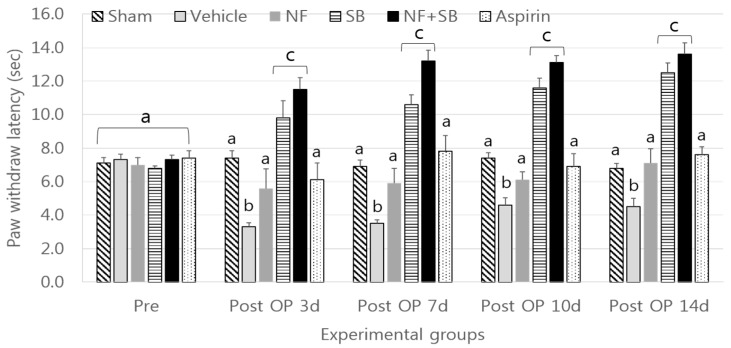
Antinociceptive effect of NF on the thermal hyperalgesia test as assessed by a hot water test at the pre, 3rd, 7th, 10th and 14th day following the nerve crush injury. Data are presented as mean ± SEM. Data were analyzed by ANOVA with Tukey’s post hoc analyses; Values with different letters are significantly different at *p* < 0.05.

Thermal hyperalgesia was performed for behavioral experiments. This is a method of observing the avoidance reaction when the harmful heat stimulus, excites the nociceptor and causes the pain. The paw withdraw reaction is a rapid response to painful thermal stimuli that is a direct indicator of the nociceptive threshold. As a positive control, aspirin was used as a typical analgesic that is used as symptomatic relief of mild to moderate pain [26,27,28]. It has anti-inflammatory and anti-thrombotic effects but can cause gastrointestinal bleeding when an overdosed is taken [29,30]. In this experiment, there was a significant antinociceptive effect in the thermal hyperalgesia test in the aspirin group as compared with the vehicle group on the at 7th, 10th, 14th day. This study also showed that latency of the paw withdraw reaction was delayed with a statistically significant difference (*p* < 0.05) in the NF, SB, and NF + SB treated groups as compared to the vehicle group. Also, in the NF group treated with the TRPV1 antagonist (SB366791), significantly delayed paw withdraw reaction (*p* < 0.05) were obtained on the 3rd, 7th, 10th, and 14th day as compared with the vehicle (Figure 1). It showed additive effect of NF when administered with TRPV1 antagonist when compared with TRPV1 antagonist alone or NF alone at the 3rd, 7th, 10th, 14th day.

### 3.2. Analysis of Inflammatory Markers

**Figure 2:**
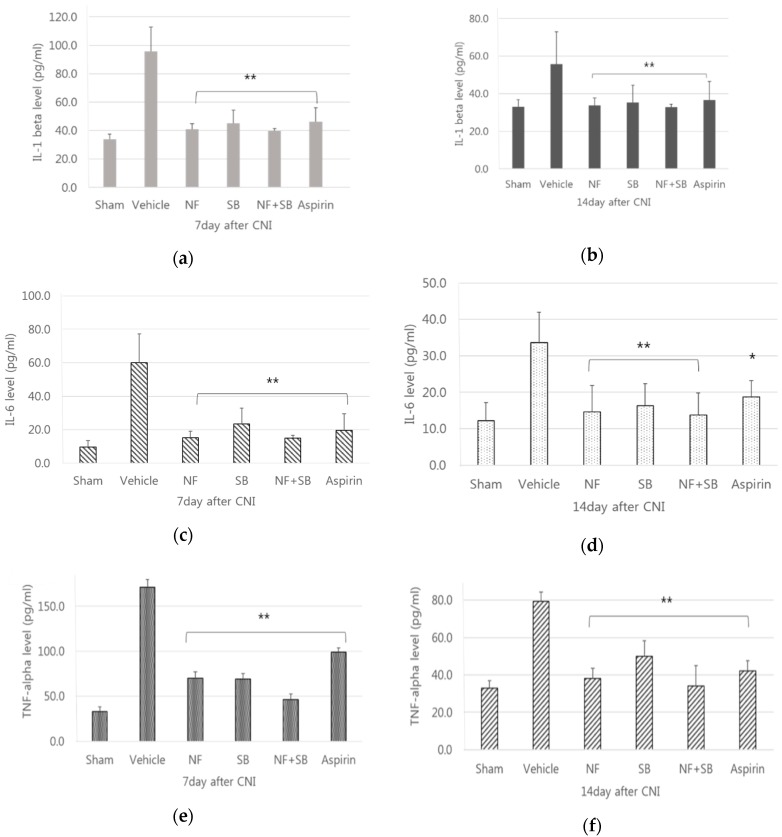
NFE (*Nypa fruticans* Wurmb extracts) affects the levels of (**a**) IL-1β, (**c**) IL-6, and (**e**) TNF-α at the 7th day and the levels of (**b**) IL-1β, (**d**) IL-6 and (**f**) TNF-α at the 14th day in the serum of the sciatic crush injury rats. In the *Nypa fruticans* (NF) and NF + SB treated groups, the levels of (**a**) IL-1β, (**b**) IL-6, and (**c**) TNF-α were significantly lower than those in the vehicle group. Data are expressed as the mean ± SEM. (*; *p* < 0.05, **; *p* < 0.01 vs. vehicle group, Kruskal–Wallis test followed by a post hoc Mann–Whitney U test).

Initial investigation of TRPV1 modulation of pro-inflammatory cytokines in vivo has been centered on the reactivity of pain promoting the release of pro-inflammatory cytokines, including IL-6, TNF-α, and IL-1β [31,32]. TRPV1 also modulated the occurrence and development of numerous neurological disorders by regulating the inflammatory cytokines in the central nervous system (CNS). These studies further illustrated that TRPV1 regulates the synaptic transmission and plasticity in neurons and interacts with pro-inflammatory cytokines in the process of inflammatory neurological diseases [33]. The serologic evaluation confirmed the pro-inflammatory factors; IL-1β, IL-6, and TNF-α. Pro-inflammatory cytokines, including IL-1β, IL-6, and TNF-α were measured in the NF, SB, NF + SB treated groups on the 7th, and 14th days. The levels of IL-1β, IL-6, and TNF-α in NF (40.9 ± 3.9, 15.1 ± 6.4, 70.1 ± 18.3) SB (45.0 ± 9.2, 23.5 ± 3.3, 69.2 ± 31.5), NF + SB (39.8 ± 1.6, 15.0 ± 3.4, 46.5 ± 33.6) treated groups at the 7th day were significantly lower than those in the vehicle group (95.7 ± 17.2, 60.0 ±7.6, 171.1 ± 53.8; *p* < 0.05) (Figure 2a,c,e). The levels of IL-1β, IL-6 and TNF-α in NF (33.7 ± 4.0, 14.6 ± 11.3, 38.1 ± 19.0), SB (35.3 ± 5.1, 18.3 ± 14.0, 50.0 ± 28.4), NF + SB (32.8 ± 2.5, 13.8 ± 11.0, 34.1 ± 37.8) treated groups at the 14th day were significantly lower than those in the vehicle group (55.7 ± 7.7, 33.6 ± 21.3, 79.3 ± 16.9; *p* < 0.05) (Figure 2b,d,f). 

### 3.3. The COX2 Expression in Sciatic Crush Injury Models

**Figure 3:**
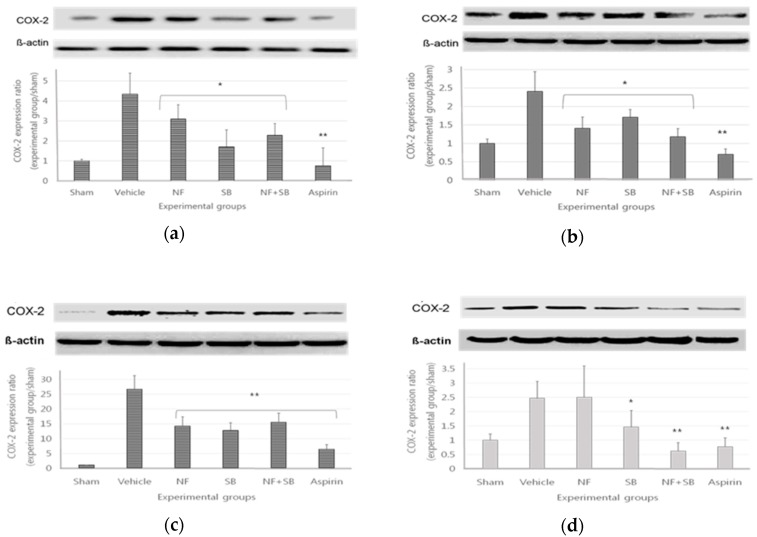
NFE down-regulates the expression of COX2 in the sciatic nerve and the L4–L6 spinal cord at the 7th (**a**,**c**) and 14th days (**b**,**d**) following the crushed nerve injury. The density data are the mean ± SEM values of experiments. (*; *p* < 0.05, **; *p* < 0.01 vs. vehicle group, Kruskal–Wallis test followed by a post hoc Mann–Whitney U test).

Cyclooxygenase 2 (COX2), a key enzyme in the biosynthesis of prostaglandins, consequently enhanced the synthesis of prostaglandin and promoted an inflammatory response. Recent evidence suggests that the expression of COX2 may be involved in the development of neuropathic pain after nerve injury [34]. At the 7th and 14th days after surgery, sciatic nerve and L4-L6 spinal cord samples were taken for a western blot analysis. In the NF (*p* < 0.05), SB (*p* < 0.05), and NF + SB (*p* < 0.01) treated groups, the COX2 expression was significantly suppressed on the 7th (Figure 3a,c) and 14th days (Figure 3b,d) as compared to the vehicle group. It is noteworthy that NFE has anti-inflammatory properties in sciatic crush injury models.

### 3.4. The TRPV1 Expression in Sciatic Crush Injury Models 

**Figure 4:**
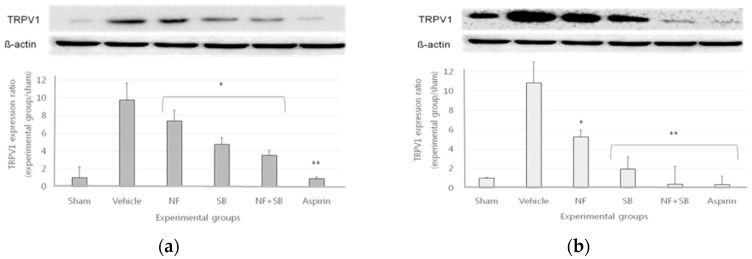

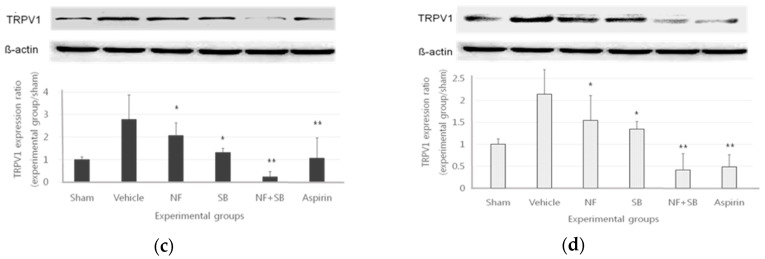
NFE down-regulates the expression of TRPV1 in the sciatic nerve at 7th day (**a**,**c**) and the L4-L6 spinal cord at 14th days (**b**,**d**) following the crushed nerve injury. The density data are the mean ± SEM values of experiments. (*; *p* < 0.05, **; *p* < 0.01 vs. vehicle group, Kruskal–Wallis test followed by a post hoc Mann–Whitney U test).

Sciatic nerve and L4-L6 spinal cord samples were analyzed using a western blot analysis on the 7th and 14th days after the CNI surgery. In the vehicle group, the expression of TRPV1 increased as compared to the sham group. On the other hand, the expression of TRPV1 showed a suppressive effect on the sciatic nerve and spinal cords of the NF treated group (*p* < 0.05) and SB treated group (*p* < 0.05) at the 7th (Figure 4a,c) and 14th days (Figure 4b,d). Also, the NF + SB (*p* < 0.01) treated group showed significantly inhibition of TRPV1 expression. 

## 4. Discussion

Neuropathic pain (NP) is a complex network of several molecular processes, including nitro-oxidative stress, immune response, and TRP channels activation, among others. NP is not only promoted by direct injury to neurons but also by TRP channels mediating damage in surrounding tissue [35]. However, how these actors and other factors (e.g., sodium channels, acid-sensing ion channels, and synaptic receptors) are interconnected leading to noxious symptomatology remains unresolved [36]. Interactions between TRPV1 and prolonged sustained thermal hypersensitivity in oxidative stress-induced inflammatory hyperalgesia of mouse hind paws have been reported [37]. Current studies have strongly supported the important role of TRPV1 mediating the interchange and bidirectional contact of inflammatory cytokines [38]. Additionally, there is a direct relationship between increased levels of reactive oxygen species (ROS) and inflammatory hyperalgesia [39]. Also, a peripheral nerve injury leads to an inflammatory response and the rapid production and release of cytokines such as interleukin-1 and tumor necrosis factor. These can cause the infiltration of neutrophills and proinflammatory M1 monocytes/macrophages into the distal nerve stump, which impairs the recovery of the sciatic nerve [2,40]. Inflammatory mediators including inflammatory cytokines, prostaglandins, nitric oxide (NO) and ROS, are probably implicated in acute or chronic inflammatory responses [41,42]. Neuropathic pain is associated with inflammatory cytokines in locally recruited macrophages, Schwann cells, and glial cells [43]. TRPV1 promotes an influx of Na^+^ and Ca^2+^, store-operated Ca^2+^ entry (SOCE), and neuronal cell membrane depolarization. Therefore, it directly affects cellular responses, such as morphological changes, proliferation, migration [44], phagocytic activity [45], and the secretion of inflammatory cytokines [46]. NF, which was used as a folk remedy [19,20], is a plant that is gaining more attention due to its various effects. Previous studies have reported the anti-nociception, antioxidant, and anti-inflammatory effects of NF [18]. However, there have been no studies that look at NFE relation to neuropathic injury.

This study was carried out based on the hypothesis that NFE has antinociceptive and anti-inflammatory effect by controlling the neurological function of TRPV1. In the thermal hyperalgesia test, there was a significant antinociceptive effect in the NFE treated group compared with the vehicle group at the 7th, 10th, 14th days (Figure 1). Although NFE did not show as comparable antinociceptive effects as a TRPV1 antagonist, the results of this work showed the significant antinociceptive effect of NFE similar with aspirin medication on a sciatic nerve crushing injury. Also, the expression of TRPV1 increased in the vehicle group but the expression of TRPV1 was significantly downregulated by NFE in the sciatic nerve, and L4-L6 spinal cord (Figure 3). Notably, NFE affects the expression of TRPV1 in sciatic nerve injuries. In particular, TRPV1 expression was significantly decreased NF treated with TRPV1 antagonist compared with TRPV1 antagonist. In addition, NFE showed a partially antinociceptive additive effect when administered with TRPV1 antagonist compared with TRPV1 antagonist alone. Several reports have shown the possibility of a potential analgesic to target TRPV1. To date, the functions of TRPV1 in the peripheral nervous system (PNS) were under comprehensive investigation and their potential application as a target for treating neurological disorders has been extensively demonstrated [47,48,49]. Initial investigation of TRPV1 modulation of pro-inflammatory cytokines in vivo has been centered on the reactivity of pain to promote the release of pro-inflammatory cytokines, including IL-1β, IL-6, and TNF-α [31,32]. Certainly, TRPV1 also modulated the occurrence and development of numerous neurological disorders by regulating the inflammatory cytokines in the CNS. TRPV1 has been demonstrated to reduce inflammation by modulating various inflammatory signaling pathways. Endogenous ligands of TRPV1 also lead to anti-inflammatory action via inhibition of the production of IL-6 and NO. 

Moreover, TRPV1 could regulate the expression COX-2 at the gene level of the macrophages through interfering with the upstream signaling events of LPS and IFN-γ [50]. Based on previous studies, we confirmed that the levels of TNF-α, IL-1β, and IL-6 in the NFE treated groups are significantly lower than those in the vehicle group. Additionally, NFE treated groups significantly suppressed the expression of COX-2 (Figure 3). NFE has anti-inflammatory properties relating to sciatic neuropathies. Here, NFE suppresses TRPV1 expression to reduce inflammatory factors and sciatic neuropathy, and I think it would show antinociceptive effect. This study is supported by several studies that TRPV1 is expected to act on the peripheral pain site and CNS, thereby reducing the incidence of pain and modulating neuroinflammation in regulating immune processes in the CNS [51,52]. This study suggests that ethanol extracts of NF can be distinguished from the effects of previous research about methanol extract of NF. However, further detailed studies are essential to find out the underlying mechanisms of antinociceptive activities and also to isolate the active compound responsible for those pharmacological properties [53].

## 5. Conclusions

This study showed the antinociceptive and anti-inflammatory effects of NFE and the inhibition of TRPV1 in neuropathic pain models induced by a sciatic nerve crushing injury. In addition, proper administration with TRPV1 antagonist may contribute to the treatment of sciatic neuropathy. However, the nervous system has a very complex structure and has various interactions. Additionally, it is difficult to expect a therapeutic effect due to the action of particular mechanism or part. Therefore, the mechanism underlying the effects of NFE on neuropathic injuries remains to be explored by future studies.

## 6. Patents

South Korea Patent Number: 10-2019-0004034 “Composition for preventing and treating of neuropathic pain containing *Nypa friticans* Wurmb extract”.
